# The Role of By-Products of Fruit and Vegetable Processing for the Dietary Treatment of Cardiovascular Risk Factors: A Narrative Review

**DOI:** 10.3390/antiox11112170

**Published:** 2022-11-01

**Authors:** Isabela Ribeiro Grangeira Tavares, Vivian dos Santos Pinheiro, Patrícia Marques Lisboa Aroso de Castro, Isabelle Barbosa Reis, Gustavo Vieira de Oliveira, Thiago Silveira Alvares

**Affiliations:** 1 Nutrition and Exercise Metabolism Research Group, Multidisciplinary Center, Nutrition Institute, Federal University of Rio de Janeiro, Macaé 27979-000, Brazil; 2 Multidisciplinary Center, Institute of Medical Sciences, Federal University of Rio de Janeiro, Macaé 27979-000, Brazil; 3 Multidisciplinary Center, Food and Nutrition Institute, Federal University of Rio de Janeiro, Macaé 27979-000, Brazil

**Keywords:** nutrition, food and health, food science, functional food

## Abstract

Polyphenols-rich food has been utilized to induce a positive effect on human health. Considering that fruit and vegetable by-products (seeds, pomace, and peels) are sources of polyphenols, previous studies have investigated the effect of dietary supplementation with food by-products on cardiometabolic disorders, such as high fasting blood glucose, dyslipidemia, and obesity. Endothelial dysfunction has also been considered a cardiometabolic parameter, given that it precedes cardiovascular disease. However, there is a scarcity of narrative reviews reporting the effect of food by-product supplementation on cardiometabolic disorders in animal and human clinical trials. In this sense, the present narrative review aims to investigate the impact of fruit and vegetable by-product supplementation on cardiometabolic disorders in humans and animals, exploring the possible mechanisms whenever possible. Research articles were retrieved based on a search of the following databases: PubMed, ScienceDirect, and Google Scholar using the following keywords and synonyms combined: (“fruit by-products” or “food waste” or “pomace” or “bagasse” or “seeds” or “waste products”) AND (“heart disease risk factors” or “endothelial dysfunction” or “atherosclerosis”). It was shown that fruit and vegetable by-products could efficiently improve cardiometabolic disorders in patients with chronic diseases, including hypertension, type II diabetes mellitus, and dyslipidemia. Such effects can be induced by the polyphenols present in food by-products. In conclusion, food by-product supplementation has a positive effect on cardiometabolic disorders. However, further studies investigating the effect of food by-products on cardiometabolic disorders in humans are still necessary so that solid conclusions can be drawn.

## 1. Introduction

Metabolic disorders such as obesity, dyslipidemia, type 2 diabetes mellitus (T2DM), and hypertension precede cardiovascular diseases (CVDs), the main cause of death worldwide [1]. These metabolic disorders contribute to the reduction of nitric oxide (NO) bioavailability, resulting in endothelial dysfunction, a condition that precedes cardiovascular events [2,3]. Therefore, metabolic disorders have drawn the attention of researchers seeking to develop nutritional strategies to reduce cardiometabolic parameters [4]. Many studies have recommended ingesting fruits and vegetable-derived polyphenols to prevent cardiometabolic diseases, given their effect in modulating glucose and lipid metabolism, among other factors [5].

Polyphenols are bioactive compounds produced in the secondary metabolism of plants, and possess aromatic rings in their chemical structure. About 8000 phenolic structures have been identified and classified into several subclasses: flavonoids (flavan-3-ols, flavonols, flavones, isoflavones, flavanones and anthocyanins) and non-flavonoids (stilbenes, phenolic acids and lignans) [6]. Although it is well-known that polyphenols supplementation can modulate metabolic pathways regulating fasting plasma glucose and lipids, inflammatory response, and scavenging reactive oxygen species (decreasing oxidative stress), and can increase NO bioavailability [7], the impact of food by-products-derived polyphenols on metabolic parameters needs to be better explored.

It is important to elucidate the effect of food by-product-derived polyphenols on human health since polyphenol content can be affected by adverse environments, such as low pH, light, and temperature, all of which could reduce their bioactive capacity. Considering that the food by-products are often treated as waste after the industry utilizes fruit and vegetable pulp, the polyphenol content in food by-products could be decreased due to inappropriate handling [7]. To address this issue, it would be important to investigate the impact of food by-products-derived polyphenols ingested on alleviating cardiovascular risk factors (high fasting glucose, dyslipidemia, hypertension), including endothelial function, which plays a crucial role in the development of CVDs.

Additionally, it is noteworthy that the added nutritional value of food by-products (with polyphenols, fiber, and vitamins) would be economically attractive since the food industry could utilize food by-products to elaborate microencapsulated powder to be dissolved in water as a supplement, used as an ingredient to prepare bread, cookies and pancakes, etc. [8]. Agro-industries annually produce a large number of food by-products while processing fruits and vegetables, which are disposed of improperly in the environment, causing a negative impact [9]. Thus, it would be interesting to review and explore the impact of food by-product ingestion on human cardiometabolic health so that nutritional value can be added by these by-products and to minimize their waste.

The main goal of the present narrative review is to report the effect of food by-products derived from fruits and vegetables on cardiometabolic disorders, such as high fasting blood glucose, dyslipidemia, obesity, and hypertension. Moreover, we explore the possible mechanism by which food by-products can improve cardiovascular risk factors.

## 2. Methods

A search of the scientific literature was performed to investigate the anti-diabetic, anti-obesity, antihypertensive, hypolipidemic, and vascular effects of consuming fruit and vegetable by-products. Clinical trial articles were retrieved based on a search of the following databases: PubMed, ScienceDirect, and Google Scholar using the following keywords and synonyms combined: (“fruit by-products” or “food waste” or “pomace” or “bagasse” or “seeds” or “waste products”) AND (“heart disease risk factors” or “endothelial dysfunction” or “atherosclerosis”). The search was restricted to papers written in the English language. Limits were applied to cover studies published in the last 10 years.

## 3. Anti-Diabetic Activity

Individuals with type 2 diabetes mellitus typically exhibit inflammation and high reactive oxygen species production [10,11]. Thus, previous studies have suggested that high ingestion of fruits and vegetables, rich in polyphenols compounds, is associated with a reduced risk of T2DM [12,13].

In this context, Amin et al. [14] observed a significant reduction in blood glucose in rats supplemented with an aqueous extract of the bitter apple seed for 2 weeks. Improved insulin resistance and sensitivity have been reported by Lenquiste et al. [15] after supplementation with jaboticaba peel, a fruit generally considered as a Brazilian berry, in mice for 12 weeks. Pear pomace supplementation has also shown positive effects on the glucose homeostasis of obese mice. The mice supplemented with pear pomace demonstrated a lower area under the glucose curve and HOMA-IR than the control group (which did not receive the pear pomace) [16]. Rodríguez-González et al. [17] investigated the effect of peach by-product supplementation on insulin resistance and serum glucose levels in rats fed a high-fat and high-fructose diet for 18 weeks. The authors found that the peach juice by-product supplementation produced lower serum glucose levels. In addition, the peach by-product group showed reduced HOMA index (improving insulin resistance) values compared to animals fed a high-fat and high-fructose diet. This effect was attributed to caffeoylquinic, p-coumaroylquinic, 4-acid feruloylquinic acids, and kaempferol (Table 1).

In humans, a previous study has investigated the effect of jaboticaba peel ingestion on insulin blood glucose levels. It was shown that the jaboticaba peel ingestion reduced insulin levels and the area under the plasma glucose and insulin curve [18]. These findings may be explained by the presence of phenolic compounds in jaboticaba peel, which has been demonstrated to inhibit the action of two membrane transporters in the gut (SGLT1 and GLUT2), decreasing glucose absorption and, consequently, the postprandial response [18].

A previous study has investigated the effect of 8 g polyphenol-rich grape pomace (a by-product generated during wine preparation) ingestion on cardiometabolic parameters (insulin levels, HOMA index, etc.) for 6 weeks [19]. It was demonstrated that grape pomace ingestion reduced insulin levels and improved the HOMA index [19]. Moreover, Kitada et al. [20] showed that supplementation with 5 mg/day of piceatannol (derived from passion fruit seeds) for 8 weeks in eutrophic and overweight individuals resulted in a significant decrease in fasting serum insulin levels in overweight men (from 8.3 to 6.7 µU/mL).

In contrast, Pérez-Ramírez et al. [21] evaluated the acute effect of 10 g of grape pomace ingestion associated with pomegranate pomace (in 250 mL of commercial apple-flavored water with non-caloric sweeteners) in individuals with abdominal obesity (diameter ≤94 cm for men and ≤80 cm for women) after ingesting a solution of 75 g of glucose. It was found that grape pomace/pomegranate ingestion did not affect blood glucose and insulin levels. It was also shown that the supplementation did not affect the antioxidant capacity of plasma and urine, suggesting that a single dose of grape pomace/pomegranate does not seem to be effective in controlling blood glucose and insulin levels in obesity [21] (Table 2).

**Table 1:** Summary of the studies included in the review that investigated supplementation using fruit or vegetable-based by-products in animal studies.

Study	Species	Food	Bioactive Compounds	Intervention	Sample	Primary Outcome
Amin et al. [14]	*Citrullus colocynthis*	Bitter apple	Amino acids, complex B vitamins, flavonoids, phenols, alkaloids, tannins, glycosides, triterpenoids and saponins	Seed or control 1 and 2 mL/kg/day 14 days	N = 48; Rats (males); 6–8 weeks old	↓ BG ^†^
Andrade et al. [22]	*Myrciaria cauliflora*	Jaboticaba	Ellagic acid	Peel or control 0.012; 0.12; 0.24; 0.48 and 0.96 mg/kg	N = 6, Rats (male)	↑ AVC ↔ HR ↓ MAP ↑ Relaxation endothelial ^†^
Araújo et al. [23]	*Myrciaria cauliflora*	Jaboticaba	Fibers and anthocyanins	Peel or control 7%, 10% and 15%/meal 4 weeks	N = 35; Rats (male)	↓ BG ↑ HDL-c ↓ TC ↓ TG ^†^
Bajerska et al. [24]	*Vaccinium macrocarpon*	Cranberry	Tocochromanols, flavonols, anthocyanins and fiber.	Pomace or control 3%/meal 8 weeks	N = 40; Rats(male); 56 days old	↑ FRAP ↑ GSH ↓ TBARS ↓ TG ↓TC ^†^
Benítez et al. [25]	*Allium cepa* L.	Onion	Fiber, xylose, galactose, rhamnose, arabinose and mannose	Pomace or control 10%/meal 4 weeks	N = 10; Rats (female); 6 weeks old	↑ HDL-c ↓ TC ↓ TG ^†^
Chang et al. [16]	*Pyrus* L.	Pear	Phenolic acid, flavonoids, stilbenes, tannins, carotenes and xanthophylls.	Pomace 8%/meal 5 weeks	N = 32; Rats (male); 8 weeks old	↓ BW ↓ HOMA-IR ↓ LDL-c ↓ OGTT ↓ TC ^†^
Cherrad et al. [26]	*Olea europaea*	Olive	Coumaric acid, caffeic acid, ferulic acid, oleuropein and hydroxytyrosol.	Pomace or placebo 7.5%/meal 4 weeks (28 days)	N = 12; Rats (male); diabetics	↓ BG ↑ CAT ↑ GSH ↑ GSH-Px ↓ HbA1c ↑ SOD ↓ TBARS ↓ TC ↓ TG ^†^
Del Pino-García et al. [27]	*Vitis Vinifera* L.	Grape	Anthocyanin, proanthocyanin and catechin	Pomace or control 300 mg/kg/day 4 weeks	N = 20; Rats; hypertensive and normotensive; 12 week old	↑ eNOS ↓ MDA ↑ NO ↓ SBP ↑ SOD ^†^
Dragano et al. [28]	*Myrciaria Jaboticaba (Vell.) Berg*	Jaboticaba	Cyanidin-3-O-glucoside Delphinidin-3-O-glucoside	Peel or control 1, 2 and 4%/meal 6 weeks	N = 40; Rats (male); 21 days old	↔ BW ↔ HDL ↑ iTT ↔ TC ^†^
Gerardi et al. [29]	*Vitis vinifera* L. cv. Tempranillo	Grape	Phenolic acids, stilbenes, flavanols and flavonols	Pomace or control 300 mg/kg/day 4 weeks	N = 30; Rats (male); hypertensives and diabetics	↑ eNOS ↓ ROS ^†^
John et al. [30]	*Garcinia mangostana*	Purple mangosteen	Xanthones, procyanidins, anthocyanins and hydroxycitric acid	Rind or control 5%/meal 8 weeks	N = 48; Rats (male); 8–9 weeks old	↓ AC ↓ BW ↓ Diastolic stiffness constant ↓ Inflammatory cells ↑ Relaxation endothelial ↓ SBP ↓ TC ↓ WBFM ^†,¥^
Khanal et al. [31]	*Vaccinium angustifolium Ait.*	Blueberry	Procyanidins, anthocyanins, phenolic acids and flavonols.	Pomace or control 1.5% and 3%/meal 8 weeks	N =36; Rats (male); metabolic syndrome; 46 days old	↔ BG ↔ Insulin ↓ TC ↓ TG ↓ TWF ^†^
Kukongviriyapan et al. [32]	*Antidesma thwaitesianum*	Mamao	Anthocyanins and catechin	Pomace or control 100 and 300 mg/kg/day 3 weeks	N = 10; Rats (males)	↑ ACH ↓ DBP ↑ eNOS ↓ MAP ↓ MDA ↑ NO ↓ SBP ^†^
Lenquiste et al. [15]	*Myrciaria Jaboticaba (Vell.) Berg*	Jaboticaba	Cyanidin, gallic acid, ellagic acid and glucoside	Peel or control 2%/meal 12 weeks	N = 36; Rats (male); obese	↓ AA ↓ AT ↑ iTT ↑ HDL-c ↔ TC ^†^
Lima et al. [33]	*Platonia insignis* Mart.	Bacuri	-	Seed 25 and 50 mg/kg/day 28 days	N =36; hamsters (male); dyslipidemic	↑ HDL-c ↓ LDL-c ^†^
Osforw et al. [34]	*Citrus aurantium* L.	Orange	Fiber, flavonoids, flavanone glycosides, phenolic acids and terpenes	Albedo or control 10% and 20%/meal 2 weeks	N = 32; Rats (male)	↓ BG ↓ BW ↑ HDL-c ↓ LDL-c ↓ TC ↓ TG ↓ TL ^†^
Randriamboavonjy et al. [35]	*Moringa oleifera*	Acácia-branca	Polyphenolic, glucosinolates and isothiocyanates	Seed or control 750 mg/kg/day 4 weeks	N = 46; Rats (male); 16/50-week-old	↑ eNOS ^†^
Rodríguez-González et al. [17]	*Prunus persica* L.	Peach	Phenolic acids, lignans, flavanols and flavonols	Peel and pulp or control 230.83 mg/kg/day 18 weeks	N = 32; Rats (male); obese	↓ BG ↓ BW ↓ HOMA-IR ↔ Insulin ↓ TG ↓ TyG ^†^

AA: abdominal adiposity; AC: Abdominal Circumference; ACH: Acetylcholine; AT: Adipose Tissue; AVC: Aortic Vascular Conductance; BG: Blood Glucose; BW: Body Weight; CAT: Catalase; DBP: Diastolic blood pressure; eNOS: Endothelial Nitric Oxide Synthase; FRAP: ferric-reducing ability of plasma; GSH: Reduced Glutathione; GSH-px: Glutathione Peroxidase; HbA1c: glycosylated hemoglobin, HDL-c: High Density Lipoprotein; HOMA-IR: Insulin resistance HR: Heart Rate; iTT: Insulin Tolerance Test; LDL-c: Low-Density Lipoprotein; MAP: Mean Blood Pressure; MDA: Plasma malondialdehyde; NO: Nitric Oxide; OGTT: Oral Glucose Tolerance Test; ROS: Reactive Oxygen Species; SBP: Systolic Blood Pressure; SOD: Superoxide Dismutase; TBARS: thiobarbituric-acid-reactive species; TC: Total Cholesterol; TG: Triglycerides; TL: Total Lipid; TyG: Triglycerides and glucose index; TWF: Tissue Weight Fat; WBFM: Whole-Body Fat Mass; ↓: decrease; ↑: increase; ↔: no change; ^†^: intervention effect; ^¥^: time effect.

## 4. Anti-Obesity Activity

Obesity results from low physical activity levels associated with high caloric ingestion [46]. Obesity is associated with metabolic disorders, such as T2DM, hypertension, dyslipidemia, abnormal fasting blood glucose, and certain types of cancer; thus, obesity has been considered the fifth leading cause of death worldwide [46,47].

Dietary habits are a modifiable factor in preventing and treating obesity. Long-term ingestion of a hypercaloric diet containing high sugar and fat contributes strongly to obesity [46]. However, adequate consumption of fruit- and vegetable-derived bioactive compounds is associated with preventing cardiometabolic disorders, including obesity, given the presence of substances possessing anti-obesity properties [28].

In this context, studies have investigated the effect of fruit and vegetable by-products on managing body weight in humans and animals. For example, Osforw et al. [34] evaluated the hypocholesterolemic and hypoglycemic effects of dietary orange albedo flour (*Citrus Aurantium* L.) in hypercholesterolemic rats for six weeks. The groups of animals that consumed a diet containing 10% and 20% orange albedo flour had a significantly reduced body weight compared to the control group. Decreased food intake in the orange albedo flour group was also observed, which could explain the reduced body weight after supplementation [34].

Moreover, John et al. [30] observed the effect of purple mangosteen peel in rats fed a hypercaloric diet rich in sugar and fat for 8 weeks to induce cardiometabolic disorder. Rats fed with a hypercaloric diet developed obesity, hypertension, increased left ventricular stiffness, dyslipidemia, and fatty liver. Interestingly, the purple mangosteen peel supplementation reduced the body weight, abdominal fat depot (retroperitoneal adipose depot), and fat mass compared to the control group [30]. In addition, other cardiometabolic parameters (hypertension, dyslipidemia, etc.) were significantly reduced after supplementation [30].

In a trial involving jaboticaba fruit, Dragano et al. [28] investigated the impact of using freeze-dried jaboticaba peel flour ingestion on the body weight of rats for 10 weeks. The authors failed to show a significant effect of freeze-dried jaboticaba peel flour on reducing body weight [28]. Furthermore, Lenquiste et al. [15] observed the effect of freeze-dried jaboticaba peel and jaboticaba tea ingestion on body weight and visceral fat of rats fed with high-fat and high-fructose diet for 6 and 12 weeks, respectively. Both supplementations reduced body weight and fatty tissue after 6 and 12 weeks (Table 1). It has been suggested that phenolic compounds, including anthocyanins, present in jaboticaba may affect body weight [15].

Moreover, Khanal et al. [31] investigated the effect of extruded and non-extruded blueberry pomace ingestion on lowering total fat weight and abdominal fat in rats fed with a high-fructose diet for 8 weeks. It was reported that unextruded or extruded blueberry pomace decreased total fat weight compared to the control group. In addition, leptin levels were lower in animals supplemented with unextruded blueberry pomace in the fasting state or unextruded blueberry pomace in the fed state compared to the control group, suggesting that the supplementation can affect food intake regulation [31] (Table 1).

In humans, Kitada et al. [20] investigated the effect of 20 mg/day of piceatannol, a compound present in passion fruit (*Passiflora edulis*) seed extract, on metabolic health in overweight and non-overweight men and women for 8 weeks. No significant effect on the individuals’ body weight and body composition was observed [20] (Table 2). Moreover, a study by Kopčeková et al. [42] evaluated the short-term effect of consuming 60 mg/kg of phytonutrients in bitter apricot seeds (*Prunus Armeniaca* L.) on cardiovascular risk factors in adults with high cholesterol levels for 6 weeks. Individuals with normal total cholesterol had a reduction in body weight, body mass index, and body fat mass. In contrast, individuals with a high cholesterol level significantly increased body weight, body mass index, and visceral fat [42]. Additionally, Soltani et al. [45] reported that daily consumption of a medicinal capsule containing 500 mg of the extract of cucumber seeds (*Cucumis sativus*) for 6 weeks in adult patients with mild hyperlipidemia significantly reduced body mass index while there were no significant changes in the placebo group. When comparing both groups, the cucumber seed extract showed a significant reduction in body mass index, suggesting its use for weight loss in obese and overweight patients [45] (Table 2).

## 5. Antihypertensive Activity

Hypertension is a condition by which systolic and/or diastolic blood pressure is abnormal (systolic >135 mm Hg and diastolic >90 mm Hg) [48]. Although hypertension results from several factors, endothelial dysfunction and atherosclerosis play a critical role in hypertension [49,50]. Nitric oxide (NO) is the most important molecule in vascular tonus modulation; thus, a change in the NO bioavailability is associated with endothelial dysfunction and atherosclerosis in many clinical populations. In this context, previous studies focused on investigating the effect of food-derived polyphenols supplementation in improving vascular parameters [27,32]. It is expected that polyphenols’ anti-inflammatory and antioxidant properties could enhance NO bioavailability, resulting in improved blood pressure [7].

Previous studies have demonstrated that by-products obtained from fruits and vegetables, such as pomace, seeds, and peels, may have antihypertensive effects due to their antioxidant, anti-inflammatory, and vasodilator properties, all of which could improve vascular function and decrease blood pressure [20,22,27,30,32]. The antihypertensive effects of food by-products were investigated after mamao pomace [32] and red wine pomace powder [27] supplementation in hypertensive rats. In the Kukongviriyapan et al. [32] study, male Sprague-Dawley rats underwent N ω-nitro-L-arginine methyl ester (L-NAME) administration to inhibit endothelial NO synthase (eNOS), which increased systolic blood pressure (SBP), diastolic blood pressure (DBP), and mean arterial pressure (MAP). After administration of mamao pomace combined with L-NAME, effective prevention in L-NAME-induced increase in SBP, DBP, and MAP was observed, suggesting an antihypertensive effect of mamao pomace [32]. 

In another study, Del Pino-García et al. [27] also showed an antihypertensive effect in rats after administration of red wine pomace powder in 300 mg for 4 weeks. These findings were attributed to the antioxidant effect of red wine, which affected NO production, improving vascular function and, consequently, hypertension [27]. John et al. [30] investigated in rats with hypertension and arterial stiffness the effect of the addition of 5% purple mangosteen rind (*G. mangostana*) to the high carbohydrate and high-fat diet for 8 weeks. They demonstrated a significant reduction in SBP and left ventricular diastolic stiffness, greater endothelial relaxation mediated by acetylcholine, and decreased inflammatory response due to reduced inflammatory cell infiltration. Moreover, Andrade et al. [22] reported that administration of hydroalcoholic extract of *M. cauliflora* at doses up to 0.96 in isolated pre-contracted aortas of rats induced endothelial relaxation, which increased aortic vascular conductance. The presence of substances such as Pyranocyanin B, quercetin, isoquercitrin, quercimeritrin, quercitrin, rutin, gallic acid, and ellagic acid, interferes in the functionality of endothelial cells, resulting in a reduction in blood pressure and vascular relaxation [36] (Table 1).

In humans, Kitada et al. [20] investigated the effects of 20 mg/day of piceatannol supplementation for 8 weeks on blood pressure. It was shown that piceatannol supplementation significantly reduced SBP and DBP in overweight men. However, no significant effect on endothelial function, lipids, inflammation, or oxidative stress was detected in this study [20].

## 6. Hypolipidemic Activity

Dyslipidemia is characterized by changes in serum lipoprotein concentrations, such as increased low-density lipoprotein cholesterol (LDL-c) and triglycerides, as well as decreased high-density lipoprotein cholesterol (HDL-c) [51]. Dyslipidemia can occur due to genetics, lifestyle (poor diet, smoking, alcoholism, and sedentary lifestyle), use of medications (cyclosporine, beta-blockers, and protease inhibitors), and/or metabolic diseases (T2DM and non-alcoholic steatohepatitis) [52,53].

Dyslipidemia plays an important role in CVD since hyperlipidemia is associated with the initiation and progression of atherogenesis [54,55]. Given the impact of dyslipidemia on atherosclerosis, the impact of fruit and vegetable ingestion has been widely investigated due to the presence of bioactive compounds and fiber, which could improve elevated serum lipid levels [54,56]. Food-derived bioactive compounds with potential benefits for CVD are found in fruit and vegetable skin, pomace, and seeds. Several studies have demonstrated the beneficial effects of fruit and vegetable by-product ingestion on preventing and/or treating dyslipidemia [52,56].

Previous studies have investigated the effect of food derived-by products from grapes [37,44], beetroot [43], apricot [41,42], jaboticaba [15,23], onion [25], cranberry [24], pear [16], olive [26], peach [17], blueberry [31], and bacuri [33] on serum lipids. Araújo et al. [23] investigated the effect of jaboticaba peel flour supplementation in rats fed with a hyperlipidemic diet for 4 weeks. It was shown that jabuticaba peel flour supplementation decreased total serum cholesterol and triglycerides and increased HDL-c levels compared to the control group. Similarly, Lenquiste et al. [15] found an increase in HDL-c and prevention of hepatic steatosis in obese rats after 12 weeks of jaboticaba peel flour supplementation. 

Other anthocyanin-rich fruits, such as cranberry and blueberry, have beneficial effects on plasma lipid metabolism, as demonstrated in previous studies [24,31]. Bajerska et al. [24] found a significant increase in fecal lipid excretion and a decrease in serum triglyceride levels with the addition of 3% cranberry pomace in rats fed with a high-fat diet. In addition, improved plasma antioxidant capacity and decreased lipid oxidation were found. Khanal et al. [31] reported that blueberry pomace was used in rats fed with a high-fructose diet (58% fructose). The dietary intake of 3% blueberry decreased plasma cholesterol, but there were no significant effects on postprandial plasma triglycerides [31].

Moreover, Benítez et al. [25] studied rats fed with a high-fat diet enriched with onion pomace (*Allium cepa* L.) or placebo for 4 weeks. Onion pomace attenuated serum lipid and total cholesterol increases induced by a high-fat diet. Although the author attributed such a finding mainly to the fiber content, polyphenols present in onion pomace could also slightly affect serum lipid levels [25].

Among foods that potentially benefit plasma lipoproteins, grape by-products have presented interesting results in humans. Several compounds in grape skins and seeds, such as resveratrol, phenolic acids, proanthocyanidins, fiber, and others, can affect serum lipids. Argani et al. [37] investigated the benefits of supplementing 200 mg/day of red grape seed extract or placebo for 8 weeks in individuals with hyperlipidemia. A significant increase in the serum levels of apolipoprotein AI, HDL, and paraoxonase was found in the group that received the grape extract intervention. In addition, a reduction in total cholesterol, triglycerides, and LDL levels was also observed. Furthermore, paraoxonase activity was significantly correlated with apolipoprotein AI and HDL. The authors suggested that supplementation with grape extract may increase paraoxonase activity by increasing HDL-C and apolipoprotein AI levels [37].

Similarly, Razavi et al. [44] reported reduced total cholesterol, LDL, and Ox-LDL after supplementation with 200 mg/day of red grape seed extract for 8 weeks. However, significant changes were not observed in the other parameters evaluated (triglycerides and HDL). Soltani et al. [45] investigated the effect of cucumber extract supplementation on plasma lipoproteins in individuals with hyperlipidemia. The authors investigated individuals who received 500 mg/day of cucumber seed extract or placebo for 6 weeks. A significant reduction in total cholesterol, LDL-C, triglycerides, and an increase in HDL-C were reported when compared to the placebo group [45].

## 7. Vascular Effect

Endothelial dysfunction is a condition that precedes the development of atherosclerosis and CVD [38]. Endothelial dysfunction is characterized by reduced bioavailability of NO, an important vasodilator molecule produced by endothelial cells [35]. Typically, reduction in NO bioavailability is associated with increased production of reactive oxygen species and abnormal inflammatory response [3,27,32,57]. In such circumstances, vascular endothelium is dysfunctional, which can lead to the development of CVD and death [3,20,22]. Given the need to reduce the risk of CVD, nutritional strategies have been proposed to improve vascular function (Table 1 and Table 2).

Andrade et al. [22] demonstrated that supplementation with jaboticaba peel extracts in male Wistar rats, after inducing endothelial dysfunction by administration of an inhibitor of eNOS (L-NAME), increased relaxation of the thoracic artery, suggesting a vasoactive effect of jaboticaba peel extract. Moreover, Kukongviriyapan et al. [32] investigated the effect of the pomace extract of a tropical fruit (named mamao) on L-NAME-induced endothelial dysfunction parameters and markers of oxidative stress (e.g., superoxide production). It was demonstrated that dietary supplementation witih pomace extract reduced the action of vasoconstrictors (L-NAME) and increased the eNOS expression protein compared with the control group, preventing the increase in blood pressure. The antihypertensive effect of mamao pomace was related to the suppression of superoxide production from carotid strips and enhanced eNOS protein expression and NO bioavailability. Del Pino-García et al. [27] also observed that polyphenol-rich powdered red wine pomace supplementation for 4 weeks increased eNOS enzyme expression in aortic gene expression in Wistar-Kyoto rats, resulting in a decrease in blood pressure. 

Gerardi et al. [29] have also investigated grape pomace supplementation in hypertensive and diabetic rats. This study observed that the supplementation reduced the thickness of the thoracic and abdominal aortic wall (characteristics of arterial stiffness) compared to the placebo group. Furthermore, supplementation reduced the production of reactive oxygen species in hypertensive and diabetic rats compared to placebo. An increase in eNOS protein expression levels was also observed in the hypertensive rats in grape pomace compared to the control group [29].

John et al. [30] investigated the effect of powder from mangosteen (*Garcinia mangostana*) peel supplementation in rats fed a diet with increased simple sugars and saturated fats. There was a significant improvement in SBP, left ventricular stiffness, and endothelial function after *Garcinia mangostana*. The authors reported that these responses were associated with decreased inflammatory cell infiltration and decreased collagen deposition. 

The effects of Moringa supplementation on aging-associated endothelial dysfunction have been evaluated in rats [35]. This study [35] demonstrated a reduction in endothelium-dependent relaxation of arteries in aged rats compared to young/adult rats. However, an improvement of such parameters was returned after Moringa supplementation in aged rats. Such effects were attributed to enhanced eNOS signaling, which plays a crucial role in NO synthesis in vascular endothelium (Table 1). 

In humans, few studies have investigated the effect of food by-product ingestion on endothelial dysfunction. Kitada et al. [20] investigated the effect of passion fruit seed extract on endothelial function (by the technique of flow-mediated dilation—FMD) in adults and elderly of both sexes. However, no significant difference between the groups of supplementations was observed. Similarly, Fan et al. [39] also evaluated endothelial function (by FMD response) in obese individuals of both sexes. This acute study investigated the consumption of different portions of watermelon (peel, pulp, and seeds); it was observed that consumption of these portions of the fruit in meals did not lead to significant differences in endothelial function (Table 1).

In addition to these by-product sources, chardonnay seed (rich in polyphenols, fibers, and grape seed oil) supplementation has also been investigated on human endothelial function. Corban et al. [38] evaluated endothelial function in hospitalized adults and elderly individuals with endothelial dysfunction using the reactive hyperemia-peripheral arterial tonometry (RH-PAT) technique. These individuals consumed the chardonnay seed supplement rich in polyphenols or without polyphenols. The authors reported that both supplements (rich and free from polyphenols) increased RH-PAT, suggesting that other components beyond polyphenols can be associated with improved vascular function (Table 2).

## 8. Conclusions

This study shows that food by-products can improve cardiovascular health-related parameters/markers in patients and animals. The anti-diabetic, anti-obesity, antihypertensive, hypolipidemic, and endothelial function effects seem to be mainly attributable to bioactive compounds, mainly polyphenols, in the peels, seeds, and pomace. These findings support using food by-products, currently discarded in the environment, to alleviate cardiometabolic parameters (high fasting glucose, dyslipidemia, hypertension, etc.). However, further studies investigating the effect of food by-product supplementation in humans are still needed.

## Figures and Tables

**Table 2:** Summary of the studies included in the review that investigated the supplementation of fruit or vegetable-based by-products in human studies.

Study	Species	Food	Bioactive Compounds	Intervention	Sample	Primary Outcome
Annunziata et al. [36]	*Vitis Vinifera Aglianico*	Grape	Stilbenes, phenolic acids, flavanols, favonols, and anthocyanins	Pomace (Taurisolo^®^) or placebo 400 mg/twice day 8 week	N = 216; age: 18–75 years; with BMI ≥ 18.5 kg/m²	↓ D-ROMs ↓ oxLDL ↓ TMAO ^¥^
Argani et al. [37]	*Vitis vinifera* L.	Red Grape	Proanthocyanidin	Seed extract or placebo 200 mg/day 8 weeks	N = 70; age: 21–64 years; with hyperlipidemia	↑ HDL-c ↓ LDL-c ↓ TC ↓ TG ↓ PON ^†^
Corban et al. [38]	*Vitis Vinifera Chardonnay*	Grape	Polyphenols, fibers and oil.	Seed extract or placebo 4.8 g/day 8 weeks	N = 89; age: ≥18 years; with cardiovascular risk factors	↔ RH-PAT ↓ TG ^†,¥^
Fan et al. [39]	*Citrullus lanatus*	Watermelon	L-citrulline and arginine	Rind, flesh, seeds or control 100 kcal/meal acute	N = 6; age: ≥18 years; with a overweight/obese (BMI ≥ 25 kg/m²)	↔ FMD
Han et al. [40]	*Vitis Vinifera* and *S. chinensis Baillon*	Grape and Omija fruit	Resveratrol, schizandrin and flavonoids.	Pomace and extract or placebo 342.5 + 57.5 mg (low dose) or 685 + 115 mg (high dose)/twice day 10 weeks	N = 76; age: 30–70 years; with BMI ≥ 23 kg/m²	↑ apo A1 ↓ GR ↓ GSH-Px ↓ H_2_O_2_ ↓ IL-1b ↓ LDL-c ↓ Lpa ↓ nonHDL-c ↓ SOD ↓ TBARS ↓ TC ^†,¥^
Kitada et al. [20]	*Passiflora edulis*	Passion fruit	Piceatannol	Seed extract or placebo 20 mg/day 8 weeks	N = 39; age: 20–70 years old; group with BMI < 25 or ≥ 25 kg/m²	↓ BP ↓ insulin ↓ HOMA-IR ↓ HR ↔ FMD ^†,¥^
Kopčeková et al. [41]	*Prunus armeniaca* L.	Bitter apricot	Fiber, fatty acids- oleic, linoleic	Seeds or control 60 mg/kg 12 week	N = 12; age: 20–60 years; healthy adult	↓ LDL-c ↓ TC ↓ TG ^¥^
Kopčeková et al. [42]	*Prunus armeniaca* L.	Bitter apricot	Fiber, fatty acids- oleic, linoleic	Seeds or control 60 mg/kg 6 weeks (42 days)	N = 34; age: 20–60 years, with hyperlipidemic	↓ BMI ↓ BW ↓ LDL-c ↓ TC ^¥^
Oliveira et al. [43]	*Beta Bulgaris*	Beetroot	Vitexin-2-O-rhamnoside	Leaves and stalk juice or placebo 32 mg (low dose) and 77.5 mg (high dose)/dose acute	N = 13; age: 20–59 years; with dyslipidemia	↓ GPx ↓ MDA ^¥^
Pérez-Ramírez et al. [21]	*Vitis Vinifera* L. and *Punica granatum* L.	Grape and Pomegranate	Hydrolyzable polyphenols and proanthocyanidins	Pomace or control 10 g (1:1) in 250 mL of a commercial beverage 2 weeks	N = 20; age: 40–60 years; abdominal obesity	↔ BG ↔ HOMA-beta ↔ HOMA-IR
Ramos-Romero et al. [19]	*Vitis vinifera* L. cv. Tempranillo	Grape	Insoluble fiber and proanthocyanidins	Pomace or placebo 8 g/day 6 weeks	N = 49; age: 18–70 years; with cardiometabolic risk	↓ Insulin ↓ HOMA-IR ^¥^
Razavi et al. [44]	*Vitis vinifera* L.	Red Grape	Proanthocyanidins	Seed extract or placebo 200 mg/day 8 weeks	N = 42; age: 21–64 years; with dyslipidemia.	↓ LDL-C ↓ ox-LDL ↓ TC ^¥^
Soltani et al. [45]	*Cucumis sativus*	Cucumber	Polyphenols, carotenes, alkaloids, steroids, amino acids, fibers, saponins and oil.	Seed extract or placebo 500 mg/day 6 weeks	N = 47; age: ≥18 years; with hyperlipidemic	↓ BMI ↓ TC ↓ TG ↑ HDL-c ↓ LDL-c ^†,¥^

apo A1: Apolipoprotein A1; BG: Blood Glucose; BP: Blood Pressure; BW: Body Weight; BMI: Body Mass Index; D-ROMs: Reactive Oxygen Metabolites; FMD: Flow-Mediated Dilatation; GR: Glutathione Reductase; GSH-Px: Glutathione Peroxidase; HDL-c: High Density Lipoprotein; HOMA-beta: Homeostasis model assessment for β-cell function; HOMA-IR: Insulin Resistance; HR: Heart Rate; IL: Interleukin; H_2_O_2_, Hydrogen Peroxide; LDL-c: Low-Density Lipoprotein; Lpa: Lipoprotein (a); non-HDL-c: non High Density Lipoprotein; oxLDL: Oxidized Low-Density Lipoproteins; PON: Paraoxonase; RH-PAT: Reactive Hyperemia-Peripheral Arterial Tonometry; TBARS: Thiobarbituric Acid-Reactive Substances; TC: Total Cholesterol; TG: Triglycerides; SOD: Superoxide Dismutase; TC: Total Cholesterol; TMAO: Trimethylamine-N-oxide; ↓: decrease; ↑: increase; ↔: no change; ^†^: intervention effect; ^¥^: time effect.
